# Resilience of terrestrial and aquatic fauna to historical and future wildfire regimes in western North America

**DOI:** 10.1002/ece3.8026

**Published:** 2021-08-30

**Authors:** Henriette I. Jager, Jonathan W. Long, Rachel L. Malison, Brendan P. Murphy, Ashley Rust, Luiz G. M. Silva, Rahel Sollmann, Zachary L. Steel, Mark D. Bowen, Jason B. Dunham, Joseph L. Ebersole, Rebecca L. Flitcroft

**Affiliations:** ^1^ Environmental Sciences Division Oak Ridge National Laboratory (ORNL) Oak Ridge TN USA; ^2^ U.S. Department of Agriculture Pacific Southwest Research Station Davis CA USA; ^3^ Flathead Lake Biological Station The University of Montana Polson MT USA; ^4^ School of Environmental Science Simon Fraser University Burnaby BC Canada; ^5^ Civil and Environmental Engineering Department Colorado School of Mines Golden CO USA; ^6^ Institute for Land, Water and Society Charles Sturt University Albury NSW Australia; ^7^ Department of Civil Environmental and Geomatic Engineering Stocker Lab Institute of Environmental Engineering ETH Zurich Zürich Switzerland; ^8^ Department of Wildlife, Fish, and Conservation Biology University of California Davis Davis CA USA; ^9^ Department of Environmental Science, Policy and Management University of California Berkeley CA USA; ^10^ Thomas Gast & Associates Environmental Consultants Arcata CA USA; ^11^ U.S. Geological Survey, Forest and Rangeland Ecosystem Science Center Corvallis OR USA; ^12^ Center for Public Health and Environmental Assessment Pacific Ecological Systems Division U.S. Environmental Protection Agency Corvallis OR USA; ^13^ U.S. Department of Agriculture Forest Sciences Laboratory Corvallis OR USA

**Keywords:** historical fire regime, life history, metapopulation, phenology, pyrodiversity, resilience, western North America, wildfire disturbance, wildlife

## Abstract

Wildfires in many western North American forests are becoming more frequent, larger, and severe, with changed seasonal patterns. In response, coniferous forest ecosystems will transition toward dominance by fire‐adapted hardwoods, shrubs, meadows, and grasslands, which may benefit some faunal communities, but not others. We describe factors that limit and promote faunal resilience to shifting wildfire regimes for terrestrial and aquatic ecosystems. We highlight the potential value of interspersed nonforest patches to terrestrial wildlife. Similarly, we review watershed thresholds and factors that control the resilience of aquatic ecosystems to wildfire, mediated by thermal changes and chemical, debris, and sediment loadings. We present a 2‐dimensional life history framework to describe temporal and spatial life history traits that species use to resist wildfire effects or to recover after wildfire disturbance at a metapopulation scale. The role of fire refuge is explored for metapopulations of species. In aquatic systems, recovery of assemblages postfire may be faster for smaller fires where unburned tributary basins or instream structures provide refuge from debris and sediment flows. We envision that more‐frequent, lower‐severity fires will favor opportunistic species and that less‐frequent high‐severity fires will favor better competitors. Along the spatial dimension, we hypothesize that fire regimes that are predictable and generate burned patches in close proximity to refuge will favor species that move to refuges and later recolonize, whereas fire regimes that tend to generate less‐severely burned patches may favor species that shelter in place. Looking beyond the trees to forest fauna, we consider mitigation options to enhance resilience and buy time for species facing a no‐analog future.

## Graphical abstract

Wildfires in many western North American forests are becoming more frequent, larger, and more severe due to changes in climate and past fire suppression. We propose a new life history framework for wildlife response to frequency, intensity, and patch size of wildfire disturbances. Finally, we consider forest management strategies that might enhance the resilience of wildlife under transient future conditions.

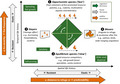

## 1 INTRODUCTION

As a natural disturbance, wildfire has shaped ecosystems of western North America. Much has been written about the feedbacks through which vegetative communities are shaped by (and shape) wildfire regimes. Conceptualizing how faunal communities will respond to nonstationary wildfire regimes is so complex that it is almost beyond comprehension. Because we know more about vegetation, it is tempting to assume that species will track their preferred vegetative communities, but we have no guarantee that present‐day communities will persist. Nor do we understand the degree to which alternative vegetation can serve functional roles required by fauna (i.e., their “substitutability”). In aquatic ecosystems, understanding habitat changes requires superimposing changes in water quality (e.g., temperature, sediment, and chemistry) from climate and wildfire, both of which can produce significant, and potentially permanent, shifts away from historical conditions. Because fauna may be facing a 6th extinction (Barnosky, 2015), due in part to no‐analog future conditions (Williams & Jackson, 2007), species conservation efforts will benefit from a mechanistic approach to understanding population‐level responses of fauna to wildlife disturbance.

In this synthesis, we seek to understand how aquatic and terrestrial fauna will be influenced by shifts in wildfire regimes in western North America. We review historical and expected future trends in wildfire and projected shifts in vegetation under future climate/fire conditions. For terrestrial fauna, we review effects of wildfire regimes, including evidence for the “pyrodiversity” hypothesis (Martin & Sapsis, 1991), which suggests that a mosaic of patches with varied burn histories and characteristics (e.g., soil characteristics, fire residuals, successional stages) will promote higher biodiversity (He et al., 2019; Minnich & Chou, 1997; Winford et al., 2015). Next, we review the effects of wildfire on aquatic habitat. For aquatic fauna, we review disturbance recovery mechanisms at different temporal and spatial scales. We propose a resilience‐based life history framework to classify wildlife traits that confer resistance and ability to recover from wildfire disturbance. Finally, we review management alternatives that may increase resilience of fauna to future changes in climate drivers and wildfire regimes.

## 2 REVIEW METHODS

This review and synthesis was developed through a hybrid approach, beginning with a review that cited 235 studies developed by the 12 authors with expertise in both aquatic and terrestrial ecosystems. A number of undocumented searches contributed to the development of the initial author review. We supplemented this search with a formal search of literature published between 1945 and 2021 conducted using Web of Science and the following query: TS = ((('wildfire') AND (((('North') NEAR/1 ('America')) OR ('Canada')) OR ('US'))) AND (((((((((('life') NEAR/1 ('history')) OR ('resilience')) OR (’pyrodiversity')) OR ('invertebrate')) OR ('fish')) OR ('amphibian')) OR ('reptile')) OR ('avian')) OR ('mammal')) NOT ‘mental’). This produced 278 references with 15 overlaps. The final review and synthesis presented here cites 264 works.

## 3 HISTORICAL, CURRENT, AND FUTURE WILDFIRE DISTURBANCE

The transition from more‐open landscapes to dense forests dominated by conifers as a result of fire suppression and other factors (Coop et al., 2020; Westerling et al., 2006). Prior to European colonization, western landscapes included more meadows (wet and dry), savannas and woodlands, shrublands and chaparral (Hessburg & Agee, 2003). As climate conditions become warmer and drier, the increase in frequency, size, and severity of wildfires in western North America is redirecting vegetation along new successional trajectories (Frelich & Reich, 1999; Halofsky et al., 2020; Hessburg et al., 2019; Johnstone et al., 2010; Morris et al., 2019).

Fire regimes in many forests across western North America are changing dramatically. Warming and drying climate trends are contributing to an increase in the frequency, size, and severity of wildfire, fueled by over a century of forest encroachment into meadows (wet and dry), savannas, and woodlands, and into shrublands and chaparral (Hessburg & Agee, 2003). The frequency of large (>400 ha) wildfires in the conterminous United States increased in the mid‐1980s (Westerling et al., 2006). An eightfold increase in area burned at high severity has occurred across western US forests between 1985 and 2017 (Parks & Abatzoglou, 2020). Wildfires have also become larger and more frequent in boreal forests of western Canada and Alaska, mediated by summer drought and drying of organic soils (e.g., peat) (Kasischke & Turetsky, 2006), and by changes in forest management (Coogan et al., 2021; Hessburg et al., 2019). For historical context, we note that burned area was high in pre‐industrial times (36–86 Mha/year) declining to 5–7 Mha/year (Leenhouts, 1998) before the rise over the most‐recent recent half‐century. Lightning strikes, which now account for >80% of burned area in the United States (Abatzoglou & Williams, 2016), are projected to increase by 12% for every 1°C increase in global mean temperature, doubling by 2,100 (Romps et al., 2014).

Factors independent of climate have also contributed to observed trends in wildfire. In some ecosystems, historical fire suppression has reduced climate resilience in recent decades (Abatzoglou & Williams, 2016). Prior to 1,800, 18.2 Mha in 11 western US states burned each year (Murphy et al., 2018). By the mid‐20th century, fire suppression efforts had reduced the annual burned area, leading to an accumulation of fuel in many ecosystems (Murphy et al., 2018). In populated areas, anthropogenic ignitions and fires have increased, expanding the area burned and extending the fire season into fall (Balch et al., 2017; Safford et al., 2012). These anthropogenic shifts in wildfire disturbance regimes have the potential to induce extreme fire behavior and loss of forest (Stephens et al., 2018).

**FIGURE 1:**
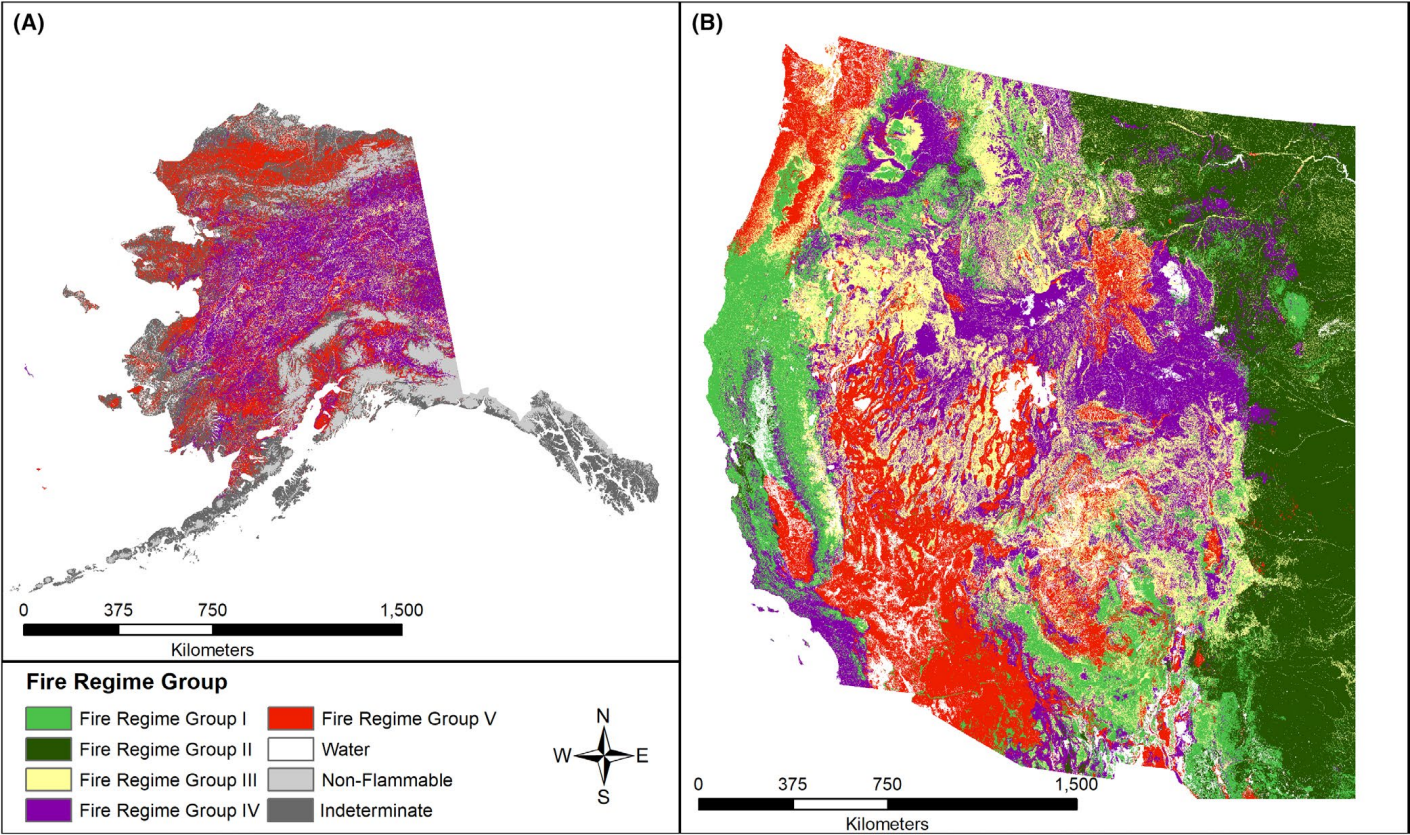
Map of historical wildfire regimes in the conterminous (a) Alaska and (b) Western United States based on LANDFIRE data. Group 1: Low‐severity fires with a 0‐ to 35‐y return interval; Group II: high‐severity fires (stand‐replacing in North America) with a 0‐ to 35‐y return interval; Group III: low‐mid severity fires with a 35‐ to 200‐y return interval; Group IV: high‐severity fires at 35‐ to 200‐y return interval; Group V: any severity fire (but typically high) at >200‐y return frequency

Changes in wildfire regime vary by region and ecosystem type (Figure 1) (Hessburg et al., 2019; Schoennagel et al., 2017). Perhaps more critical than the area burned is the severity of wildfires, which is correlated. Most of California and the southwest United States has experienced an increase in fire size and/or severity (Steel et al., 2015, 2018; Westerling, 2016; Williams et al., 2019). Burn severity has been increasing for 25% of US National Vegetation Classification communities over the past few decades, especially in regions historically characterized by frequent, low‐severity fire regimes (Group I; Figure 1). Similar increasing trends in fire size have been observed in Canada and Alaska (Kasischke & Turetsky, 2006; Kasischke et al., 2010; Wang et al., 2020). Drier conditions are expected to increase the area of boreal forest burned by 30%–500% by the end of the 21st century (Heon et al., 2014).

## 4 SHIFTS IN DOMINANT VEGETATION ASSOCIATED WITH CHANGING WILDFIRE REGIMES

Changes in feedback underlie the shifts that are occurring in western North America. Historically, an important negative feedback on wildfire recurrence intervals was the loss of fuel and subsequent delay of fires while fuel accumulated through regrowth (Seidl et al., 2016; Stevens‐Rumann et al., 2016). Fuel limitation promoted recovery of historical mixed‐coniferous forests adapted to more‐frequent, low‐severity fire. However, modern fires that escape suppression efforts often burn large areas with a high proportion of high‐severity fire (Parks & Abatzoglou, 2020). Severe or high‐severity fires, defined here as stand‐replacement fires, can favor establishment of shrubs or grasses adapted to shorter fire intervals. This new vegetation can prevent fuel limitation from acting as a negative feedback (Heon et al., 2014). When reburning occurs, positive (destabilizing) feedback can push the landscape over a tipping point leading to a non‐forest‐dominated ecosystem (Chambers et al., 2019; Coop et al., 2016; Coppoletta et al., 2016), especially in dry fuel‐limited forests at low elevations (Hessburg et al., 2019).

Whether negative feedback will be weakened or strengthened depends in part on future trends in climate and successional responses by forest and nonforest vegetation. Hessburg et al. (2019) arranged ecoregions of western North America along a spectrum from more climate‐limited (wetter, cooler) to more fuel‐limited (drier, hotter). Fuel‐limited ecosystems could transition to nonforest in response to shifting fire regimes, whereas forests with climate‐limited fire regimes may take longer to recover from fire allowing patches of grass or herbaceous cover to establish as part of the matrix (Hessburg et al., 2019).

### 4.1 Climate will mediate shifts in vegetation and fire

Disturbances, such as drought and fire, mediate transitions between vegetative states (e.g., forest/woodland, savannah, and grassland), and feedback controlling these transitions has been well described (Bowman et al., 2015; Ratajczak et al., 2014). Stabilizing negative feedback may be strong enough to keep the system from moving to a new state when perturbed (Larson et al., 2013). When this is the case, ecosystems are resilient to wildfire, returning to a preburn state over time (Burton, 2005; Rust et al., 2019). Large, repeated, or superimposed disturbances may push the ecosystem over a “tipping point” threshold onto a trajectory leading into the “basin of attraction” surrounding a different state (i.e., dominant vegetation) with its own stabilizing feedback (Hessburg et al., 2019).

A growing body of evidence suggests that fire‐driven conversion away from coniferous forest is taking place across western North America (Coop et al., 2020). Many coniferous forests in western North America are favored by more‐frequent, but smaller, moderate‐intensity fires (Coop et al., 2020). Future increases in fire size and frequency are expected to cause ecosystem shifts away from forest (Buma et al., 2013; Larson et al., 2013), starting with dry forests at the edge of their climatic tolerances (Hessburg et al., 2019). Major shifts in vegetation follow when high‐severity fires are accompanied by factors that prevent regeneration, such as a lack of proximity to seed sources, short‐interval reburning, or climatic conditions hostile to recruitment (Coop et al., 2020). In addition, competition with shrubs, annual grasses, or other flammable, fast‐growing vegetation can prevent regeneration.

Future trajectories followed by forests in western North America may vary with latitude and elevation. In boreal forests of Canada and Alaska, fuel limitation in has historically been a strong negative feedback on fire intervals at broad continental scales (i.e., across 700,000 km^2^ of unmanaged boreal forest; Heon et al., 2014; Kasischke & Turetsky, 2006; Stralberg et al., 2018). Climate limitation generally occurs in cooler, wetter settings (Hessburg et al., 2019). Negative feedback on fire may be strengthened where increased fire frequency reduces forested area and tree‐canopy cover and promotes the growth of large, fire‐tolerant trees with aerial seedbanks. Serotinous and semiserotinous conifers are affected by changing fire regimes. Fire intensity must be high enough to reach and release aerial seeds, but not high enough to destroy seeds before they reach maturity (Buma et al., 2013). Larger patches burned at high severity may favor serotiny by decreasing competition (Buma et al., 2013). In western boreal forests, increased fire sizes and frequencies are favoring hardwoods species, such as aspen, *Populus tremuloides*, with lightweight seeds that disperse long distances by wind (Whitman et al., 2019). Large fires during drought years have produced immense areas of young hardwoods (e.g., aspen) that are resistant to burning (Heon et al., 2014; Stralberg et al., 2018).

Although aspen might otherwise replace conifers (e.g., black spruce) in response to shifting fire regimes, loss of snowpack (Kretchun et al., 2020) and/or moisture stress (Barber et al., 2018) may prevent aspen from dominating and favor grasses instead. Grassland habitat is expected to expand, shifting the ecotone between grassland and forest and fragmenting Canadian forests (Barber et al., 2018). Climate projections for western boreal forests that considered aspen's low flammability and reduced fire spread predict that one‐ to two‐thirds of upland mixed and conifer forest in Alberta will be replaced by with grassland‐dominated systems by the end of the 21st century due to increased moisture stress and more‐frequent fire (Stralberg et al., 2018).

In the western United States, fire‐adapted broad‐leaf trees are expected to replace nonserotinous conifers (Morris et al., 2019), especially under Group I fire regimes (Figure 1). Broad‐leaved trees with adaptations conferring resilience to fire, such as the ability to resprout, are replacing nonresprouting, nonserotinous conifer species, aided by climate shifts that are preventing conifer regeneration (Dobrowski et al., 2015; McIntyre et al., 2015). Climate hindcasts for the southwest United States reproduced observed patterns including upward tree‐species recruitment and encroachment of scrub into semidesert grassland resulting from higher wildfire severity (Triepke et al., 2019).

High‐elevation forests are the most vulnerable to climate change. Only a small percentage are projected to remain in the current climate envelope until 2090 (Triepke et al., 2019). Fire adds to this risk; the largest increase in burned area in the western United States from 1984 to 2017 was in forests above 2,500 m (Alizadeh et al., 2021). The snowline represents a barrier to upslope fire progression (Falk et al., 2011). Future decreasing trends in snowpack suggest that high elevation areas will lose snowpack, experience drying and therefore increased wildfire risk (Eddy et al., 2018; Falk et al., 2011). An earlier start to the fire season is also associated with advanced spring snow melt (Westerling, 2016). Both drought and snowmelt timing are important proximate drivers of wildfire regimes, and harsh drought years are likely to further promote future transitions away from conifers (Harvey et al., 2016), especially in dry forests (Hessburg et al., 2005).

### 4.2 Increasing frequency of severe fire

Less‐frequent fire regimes allow time for more‐complex multilayer forests to develop, whereas more‐frequent (less‐severe) fire regimes tend to support open‐canopy forests or woodlands. Conifer regeneration may be prevented by incineration of aerial seeds by severe fires (White & Long, 2019). Coniferous forests may be pushed to a new ecosystem state when severe fires recur too frequently. Forests require a certain fire‐free interval to regenerate young trees. Once regenerated, additional time is required for saplings to grow large enough to survive subsequent fires and produce seed. Frequent stand‐replacing fires can deplete mature trees with thick bark capable of resisting future fires (Coppoletta et al., 2016; Hammett et al., 2017).

Frequent or severe fires also deplete organic soils, exposing mineral soils. In boreal forests, mineral soils promote conifer regeneration under nondrought conditions (Whitman et al., 2019) so long as seed‐limitation is not a factor (Heon et al., 2014). Paleo logical evidence suggests that extreme soil‐nutrient depletion from frequent fires can produce a negative feedback to fire by slowing regeneration and, thus, limiting fuel (Pompeani et al., 2020). Longer‐duration fires can also deplete the seed bank and promote shifts in species composition away from coniferous forest (Dale et al., 2001).

In boreal ecosystems, fire mediates a successional cycle that often starts with prolific regeneration of aspen that transitions to pine between 30 and 80 years postfire (Schieck & Song, 2006). As conifers become dominant, the forest becomes increasingly fire‐prone until fire returns (Shinneman et al., 2013). When fire intervals are long, pines persist and regenerate (Heon et al., 2014). The current increase in fire frequency is producing more‐open forests with a less‐diverse understory and less dead wood (Whitman et al., 2019).

Farther south, some lower elevation California forests are experiencing shifts toward shrubs and fast‐growing deciduous hardwood trees such as California black oak, *Quercus kelloggii* (Hammett et al., 2017; McIntyre et al., 2015; Serra‐Diaz et al., 2018). Such shifts can be self‐reinforcing (White & Long, 2019), favoring a new stable state dominated by hardwood forest maintained by more‐frequent fires. In the Southwest, some mixed oak‐pine forests are shifting to shrublands and oak‐dominated woodlands (Coop et al., 2016). Shrubs increase fire recurrence by providing fine fuels (Miller et al., 2019). Fire‐adapted shrubs also have a persistent seedbank and are therefore better able to recover from severe fire events (Miller et al., 2019).

### 4.3 Interspersion of patches burned at high severity and wildfire refuge

Another factor leading away from coniferous forests is an increase in the number and area of large, severely burned patches without proximity to fire refuge (Miller et al., 2019; Shive et al., 2018). For example, the long distance from seed sources prevents regeneration of ponderosa pine and Douglas fir, especially in dry, low‐elevation settings (Davis et al., 2019; Haffey et al., 2018; Keeley & Syphard, 2019). The spatial configuration of unburned or less‐severely burned areas (“fire refuge” or “residuals”) within the fire perimeter determines the regeneration rate and species composition of vegetation (Oliver, 1981).

Burn perimeters often contain heterogeneity in burn severity (Turner et al., 1994). These refugial areas may be absent in small fires (Turner et al., 1994), but can represent a larger fraction of intermediate‐size (Reilly et al., 2017) or large fires (Turner et al., 1994). Reilly et al. (2017) reported that patches of high‐severity burns ranged from 23% to 48% of area of fires in the Pacific Northwest, with over half in patches >100 ha in size. Particularly high proportions and large sizes of high‐severity burns occurred in cold and wet vegetation zones. In an aspen‐dominated Canadian landscape, an atypical fire burned almost the entire Kenow‐2017 fire perimeter at high severity (Eisenberg et al., 2019).

Research to understand conditions that support persistence of coniferous forests and their fauna through multiple severe fires can help to plan mitigation efforts. Fire refuge is associated with landscape features such as topographic variation (Downing et al., 2021) and wetlands, lakes, and other aquatic ecosystems (Eberhart & Woodard, 1987) that can interrupt fire and trap debris and sediment. Refuge areas in the Pacific Northwest are often found along north aspects, in valley bottoms along tributary streams and creeks near major confluences (Hessburg et al., 2015; Meddens et al., 2018). These refugial settings experience less‐frequent wildfires because of barriers to fire spread (e.g., highly dissected topography) (Camp et al., 1997; Hessburg et al., 2015; Meddens et al., 2018). Old forests can be viewed as “resistant remnant populations” sensu DeAngelis and Waterhouse (1987), resistant to wildfire because they are less flammable than younger forests (Lesmeister et al., 2019). However, refugia can burn during high‐severity fires due to high fuel loads (Kolden et al., 2017), and this risk will increase under future drier climate conditions.

The areal extent of high‐severity fires has increased relative to historical events (Whitman et al., 2019; Yocom‐Kent et al., 2015), and this trend is expected to continue as warming climate increases fuel connectivity and aridity (Halofsky et al., 2020; Reilly et al., 2017). In recent years, some burned areas have exceeded 4.05 Mha (Murphy et al., 2018), despite suppression attempts (Coop et al., 2020; North et al., 2019). Because large fires can produce a distribution of patch sizes burned at different severity (Dellasala & Hanson, 2019; Turner et al., 1994), it is important to understand how distances to refuge are affected by fire and landscape properties and the minimum size of patches that function effectively as wildlife refuge.

## 5 WILDFIRE EFFECTS ON TERRESTRIAL FAUNA IN WESTERN NORTH AMERICA

Terrestrial and aquatic fauna in forests of western North America consist of species, each of which exhibits traits that confer some degree of resilience to historical fire regimes. We developed a framework that can be used to evaluate life history strategies of aquatic and terrestrial fauna in response to fire disturbance (Box 1). The framework includes traits that influence temporal (demographic) and spatial responses and likely applies generally to fauna to other continents.

BOX 1 **Life history framework for wildlife response to wildfire disturbance**

We developed a prototype life history framework to better understand species life histories in the context of wildfire disturbance. At the scale of the individual organism, animals avoid the short‐term negative effects of fire by “sheltering in place” or moving into undisturbed areas (Lewis et al., 2016; van Mantgem et al., 2015; Thurman et al., 2020). For example, some species move to riparian habitats that serve as fire refuge, and even into water (Lyon et al., 2000; Pettit & Naiman, 2007). A common adaptation for less‐mobile species (e.g., herpetofauna, small mammals) is the use of burrows. In aquatic ecosystems, sediments and areas of river protected from silt deposition can serve as refuge for aquatic invertebrates (Mackay, 1992).

At the population level, animals have a fixed amount of energy to allocate, which induces a trade‐off among demographic traits (fecundity, early survival, and age at maturity) (Stearns, 1989). Life history allocation strategies align with gradients describing the relative importance of abiotic disturbance: predictability (resource limitation), frequency, and severity (Grime, 1977; Mims & Olden, 2012; Winemiller et al., 2015; Winemiller & Rose, 1992). Traits that increase elasticity (shorten recovery times) are associated with the ability to colonize disturbed areas, including early seral habitat or other features (e.g., snags on land, large debris in rivers) produced by fire (Robinson et al., 2014).

Below, we propose a life history framework to describe species traits align with wildfire regimes (Figure 4; Table A1). Because fecundity does not vary as much among birds and mammals as it does for fishes and other poikilotherms (Herrando‐Perez et al., 2012), we combined fecundity and juvenile survival, using the product. The proposed framework will require further analysis through ordination of species’ life history traits and the disturbance regimes that influence them, and likely applies to wildlife and fire regimes beyond North America. Such an analysis may also reveal a dependence on seasonal predictability in fire.

Understanding life history trade‐offs and how these will likely respond to future climate change is an important and open area of research. In particular, the degree of predictability in seasonal wildfire cycles will be important. Being able to predict the timing of disturbance relative to key events like reproduction will be critical to adapting to new wildfire regimes. At higher latitudes, photoperiod cues in fall tend to trigger annual reproductive development in long‐lived mammals, typically in fall, whereas mammals in lower latitudes can rely on temperature thresholds (Bronson, 2009). Of particular conservation concern are “capital‐breeding” species that store energy for infrequent breeding events when conditions are right. These species are typically associated with a high cost, such as a long breeding migration (Jager et al., 2008), and may establish breeding territories in unoccupied burned habitat (Burns, 2005) in anticipation of regenerating vegetation and other resources.

Climate change and shifts in wildfire regimes are likely to favor some life histories over others. We envision that more‐frequent fires will favor opportunistic species than less‐frequent fires, which will favor better competitors (Figure 4). Along the spatial dimension, we predict that the grain of interspersed refuge and predictability of fire disturbance will drive which spatial life histories are favored. Fire regimes that produce landscapes with more‐frequent fire and large distances to refuge may favor species adapted to low‐severity fire that shelter in place. Fire regimes that are seasonally predictable and produce intermediate‐sized patches burned at high‐severity with shorter distances to refuge may favor vagile, migratory species that integrate their activities across habitats (Figure 4). Research to refine this framework will be needed to quantify spatial resilience conferred by residuals as a function of distance, connectivity, and size, and to understand the correlations among life history traits and between traits and properties describing fire regimes.

Recovery of prefire fauna following severe wildfire depends how habitat, including vegetation, responds to disturbance and the ability of wildlife species to recolonize (Pausas, 2019) or shelter in place. To some extent, species responses to wildfire may align with their seral preferences, measured by years since burn (Nelson et al., 2008; Volkmann et al., 2020). However, generalizations based on seral stage alone are inadequate for several reasons. First, rates of vegetative succession depend on climate; patches burned 15 years previously in a colder boreal climate may resemble sub‐boreal forest 3 years postburn (Schieck & Song, 2006). Second, availability of habitat features may not follow seral gradients. For example, cover is provided both by stands of saplings and by mature forests with well‐developed understories. Third, many nonliving structural features of re‐growing patches (e.g., snag forests, litter, soil properties) influence wildlife habitat, and these depend on the severity and frequency of previous burns (Section 4.2). Fourth, successional trajectories followed by wildlife communities depend on habitat diversity as well as seral stage (Section 4.3). Finally, beyond succession of vegetation, bottom‐up recolonization of lower trophic levels is needed to rebuild food webs (Geary et al., 2019; Hammond & Theimer, 2020; Seavy & Alexander, 2014).

Habitat needs related to fire disturbance and recovery differ for terrestrial fauna with different life histories (Box 1). Small mammals, which have an opportunistic life history (Figure 4), tend to respond positively to more‐frequent, low‐ to moderate‐severity fires (Fontaine & Kennedy, 2012). Deer mice, *Peromyscus maniculatus,* are early colonizers of burned habitats and can show large increases in abundance (Converse et al., 2006; Sollmann et al., 2015). Granivores are better able to find seeds in recently disturbed open areas following less‐severe fires. However, many species (e.g., chipmunks and voles) require structural cover, such as litter or downed trees, or an understory as protection from predators (Converse et al., 2006; Sollmann et al., 2015).

Postburn canopy closure influences how many volant (flying) species respond to fire severity. Birds favoring open conditions (e.g., western bluebird, *Sialia mexicana*) respond positively to fire, whereas those favoring closed‐canopy, mesic forest habitat (e.g., hermit thrush, *Catharus guttatus*) show negative responses to wildfire (Fontaine & Kennedy, 2012). Canopy‐nesting bird species and those that forage in the foliage show significant negative responses (Fontaine & Kennedy, 2012). Cavity‐nesting birds (e.g., woodpeckers) tend to be associated with older forests (Schieck & Song, 2006).

Bat responses to wildfire are mediated by roosting habitat as well as canopy closure (Schieck & Song, 2006). Bat species with traits adapting them to foraging in open habitats tend to be associated with higher severity and more‐frequent fires, whereas those with traits consistent with clutter tolerance tend to be negatively associated with fire frequency and burn severity (Blakey et al., 2019). In dense coniferous forest, both open‐ and clutter‐adapted bats occurred more often in burned areas, with at least 35% increasing with burn severity (Steel et al., 2019). Similarly, small‐bodied bats dominate in high‐severity burned areas (Buchalski et al., 2013). Finally, riparian areas are important for bats, which respond to postfire differences in aquatic insect production (Buchalski et al., 2013).

Most ungulates, including pronghorn, bison, and mule deer and elk, benefit from browsing opportunities in early‐mid successional habitat postfire (young hardwood saplings and shrubs) (Volkmann et al., 2020). In boreal ecosystems, postfire succession has been linked to booming populations of herbivores and climate‐driven population cycles (Fox, 1978). Species often require different seral stages at different times. For example, caribou, *Rangifer tarandus*, browse in earlier stages of succession during summer, but require lichen, found in late‐successional boreal forest, as winter forage (Joly et al., 2010). As quintessential Movers (Figure 4), migratory ungulates depend on tracking dynamic resources (e.g., spring green‐up) under changing conditions (Malpeli et al., 2020).

Apex predators tend to be resilient to fire disturbance because, as Movers (Box 1) they are able to meet generalized habitat needs within large home ranges spanning multiple habitats (Geary et al., 2019) representing a wide range of years postfire (Volkmann et al., 2020). For example, American kestrels (*Falco sparverius*) continued to breed, although with lower fecundity, following fire (Dawson & Bortolotti, 2006). Predator–prey cycles (e.g., lynx–hare) occur in boreal forests of western North America, where the snowshoe hare, *Lepus americanus*, is the main prey of the Canada lynx, *Lynx canadensis*. The lynx is an endangered species that prefers forests burned 20–40 years prior (Vanbianchi et al., 2017). Lynx–hare cycles seem to be forced by periodicity in wildfire (Krebs et al., 2018) and delayed effects of winter precipitation (Yan et al., 2013). Under future climate, the juxtaposition of older and younger stands is expected to become less common for dry forests with high‐frequency, low‐severity fires (McKenzie et al., 2004).

### 5.1 Terrestrial wildlife responses to increased fire severity

Burn intensity is important for many species because it influences the availability of dead wood (snags, spars, downed logs, and coarse woody debris) used as structural habitat. Downed logs and hollows play an important role by providing a favorable microclimate, protection from predators, and nesting sites (Croft et al., 2016). These elements increase the diversity of birds and mammals postfire by providing habitat structure (Schieck & Song, 2006). For example, birds that colonize dead trees (e.g., woodpeckers) respond positively to recent fire disturbance (Stillman et al., 2019), as do saprophytic beetles. The fire‐adapted black‐backed woodpecker, *Picoides arcticus*, follows disturbances to feed on wood‐boring beetle larvae and other prey exposed by burning of tree bark (Hutto, 2008). Similarly, high‐burn severity enhances native‐bee abundance and diversity because burned habitat is crucial for wood‐cavity nesting bees and ground nesting bees use bare ground (Simanonok & Burkle, 2019).

### 5.2 Terrestrial wildlife responses to areal extent of patches burned at high‐severity and fire frequency

The increase in the size of high‐severity patches is changing forest composition and structural habitats for wildlife (Jones et al., 2016; Spies et al., 2019). Large burned patches that are devoid of large trees are less desirable as habitat for species with low gap‐crossing abilities (Viani et al., 2018) that are restricted to unburned residual areas. These unburned residual areas allow terrestrial mammals, birds, and other taxa to recolonize following fire (Perera & Buse, 2014). Although high‐severity patches can add habitat value for vagile species, they may avoid very large, burned patches. For example, California spotted owl, *Strix occidentalis occidentalis*, avoided high‐severity patches when more than 5% of their home range experienced severe fire (Jones et al., 2020). Black‐backed woodpeckers tended to nest within areas recently burned at high‐severity, but rarely in areas located more than 500 m from unburned forest or less‐severity burned edges (Stillman, Siegel, Wilkerson, Johnson, & Tingley, 2019). When large, burned areas regenerate as uniform, dense stands, wildlife diversity plummets at midsuccessional stages lacking an understory (Fox, 1983). Yet, not all populations are harmed by large fires so long as regenerating patches provide the needed resources. For example, abundances of snowshoe hare were high in regenerating even‐age stands of lodgepole pine following large fires (Hutchen & Hodges, 2019).

Increased frequency of severe fires can also trigger a shift in wildlife communities, especially when forests fail to regenerate. Roberts et al. (2015) observed a lower abundance of small mammals in frequently burned forests of the Sierra Nevada than in unburned forests. Similarly, avian communities in twice‐burned areas differ from communities in recently single‐burned areas (Fontaine et al., 2009). One reason is that wood structures that provide cover or habitat tend to be incinerated in frequently burned sites (Croft et al., 2016). In British Columbia, the proportions of small mammal species breeding in downed trees and other coarse woody debris decreases with shorter fire‐return intervals, during which time debris has accumulated (Bunnell, 1995). Frequent severe fires can also reduce larger, older fruit‐producing hardwoods and negatively impact frugivorous wildlife (Long et al., 2018). Intense, recurring fires inhibit nut production in oaks and other western hardwoods (Hammett et al., 2017). Frequent top‐killing fires may prevent fire‐adapted trees that provide food and cavities for many birds and mammals from reaching maturity (Hammett et al., 2017; Long et al., 2018).

Increased fire frequency can result in a loss of habitat for species adapted to old‐growth (late‐seral) forests, such as the California spotted owl (North et al., 2017; White & Long, 2019). These species may require, or have prey that require, a closed canopy (which facilitates spread of crown fires). Forest‐dependent species may nest in cavities or rely on mast production from stands of older trees. When fires occur, animals may experience loss of forage in severely burned patches or loss of mature trees for denning, roosting, and nesting. For example, the fisher, *Pekania pennanti*, a species associated with dense, late‐seral forest, declined after a fire in the Sierra Nevada (Sweitzer et al., 2016). Yet, there is substantial evidence that some old‐growth forest species benefit from mixed‐severity fires (Lee, 2018) (reviewed below).

### 5.3 Terrestrial wildlife responses to pyrodiversity

Fire is considered to be a key driver of the earth's biodiversity (He et al., 2019). Patterns in biodiversity can be informed by two of ecology's fundamental theories: (a) that habitat diversity leads to species diversity (Tews et al., 2004) and (b) that intermediate disturbance frequencies prevent competitive exclusion (He et al., 2019; Huston, 1979). Heterogeneity in habitat that results from fire disturbance history (i.e., burn severity, frequency, seasonality, and spatial pattern), sometimes referred to as “pyrodiversity,” has been hypothesized to have a positive association with biodiversity. This idea is supported by a growing body of research demonstrating the value of mixed‐severity fires as wildlife habitat (Taillie et al., 2018). Although the relationship between biodiversity and pyrodiversity has often focused on a single aspect of fire regime, such as time since fire, more comprehensive indices are now being used to aid in cross‐ecosystem and taxa comparisons (Hempson et al., 2018; Steel et al., 2021).

Pyrodiverse landscapes include a range of structures, resources, and seral stages that support species with different habitat needs. Pyrodiverse landscapes should support higher species diversity by facilitating coexistence of species with different preferences for wildfire disturbance (He et al., 2019; Martin & Sapsis, 1991). Habitat complementation is therefore an important mechanism to promote diversity. For example, variation in time since fire and burn severity may produce landscapes containing stands of snags used by wood‐boring beetles, woodpeckers, and bats (Steel et al., 2019; Tingley et al., 2016), shrublands inhabited by shrub‐nesting songbirds (Taillie et al., 2018), and old‐growth forests critical for some owls and meso‐carnivores (Jones et al., 2020). Similarly, forest patches containing wood falling along a spectrum from fresh to decaying also benefit different species and support higher diversity.

Diversity is a community‐level response, but at the level of individual taxa, responses to pyrodiversity vary. In a systematic review of the hypothesis, Jones and Tingley (2021) found higher support for the pyrodiversity–biodiversity hypothesis in forest/woodland ecosystems and among volant (flying) species. Specifically, studies of bats, birds, insects, and pollinators showed higher support for the hypothesis than terrestrial mammals, reptiles, and invertebrates (Jones & Tingley, 2021). Among birds, the diversity of a forest bird community increased with greater variation in burn severity in the Sierra Nevada Mountains of California, and this positive relationship increases with time since fire (Taillie et al., 2018; Tingley et al., 2016). Some avian species prefer early‐successional habitats, whereas habitat quality for others peaks at moderate‐to‐late times since fire (Taillie et al., 2018). Similarly, bat species richness in California's mixed conifer forests is highest in areas with moderate‐ to high‐burn severity and high pyrodiversity (Steel et al., 2019). Although not all studies of terrestrial mammals support the hypothesis (Jones & Tingley, 2021), infrequent fires in boreal forests of Alaska and Canada create habitat heterogeneity that favors higher wildlife diversity. Species that use late‐successional forests (>100 years since burn) include caribou that depend on lichens in winter. Because they depend on these wildlife resources (in addition to fish), indigenous Native American populations also depend on pyrodiverse landscapes (Nelson et al., 2008).

Vegetation types maintained by wildfire (i.e., forbs and grasses interspersed with the forested landscape) enhance wildlife diversity. Postdisturbance habitat provides a refuge for early‐successional species (Dominick et al., 2014) that coexist through trade‐offs between wildfire response strategies (Adam & Chesson, 2009). A wide variety of terrestrial vertebrate species rely on patches of nonconifer habitat that occur within the larger matrix of coniferous forest, including nearly 80 species documented in the Pacific Northwest alone (Hagar, 2007). For example, pollinator communities are more diverse in pyrodiverse areas of Yosemite National Park because the flowering plants on which they depend are more heterogeneous (Ponisio et al., 2016; Simanonok & Burkle, 2019). Pyrodiversity may buffer postfire pollinator communities against scarcity of floral resources caused by other disturbances such as drought (Ponisio et al., 2016).

In addition to benefiting community diversity, pyrodiverse landscapes benefit some individual species. In particular, vagile species benefit from interspersed patches with different burn histories (Buchalski et al., 2013). This has been observed in well‐studied species across the burn‐severity spectrum. The Great Gray owl, *Strix nebulosa*, prefers to nest in snags adjacent to montane meadows of California's Sierra Nevada. This state‐listed Endangered owl increased following the 2013 Rim fire, both within and beyond the fire perimeter (Siegel et al., 2019). The Northern spotted owl, *S. occidentalis caurina*, inhabits late‐seral, closed‐canopy forests that support higher densities of small mammals, including the owl's primary prey, the northern flying squirrel, *Glaucomys sabrinus* (Buchanan, 2004). However, the owl's hunting success may be higher in recently burned habitat, as long as the areal extent of the wildfire is moderate (Jones et al., 2020). And their old‐growth adapted prey, considered to have poor gap‐crossing ability, sometimes use patches with lower canopy cover (Sollmann et al., 2016). Likewise, California spotted owls respond positively to a mosaic that includes patches <36 ha of high‐severity burns as well as unburned, low, and moderate‐severity patches (Eyes et al., 2017). At the other end of the severity‐preference spectrum, adult black‐backed woodpeckers depend on fire‐killed trees and associated wood‐boring beetles. However, fledglings may prefer areas with surviving trees (Dominick et al., 2014) and adults tend to avoid nesting sites that are >500 m from the closest patch of live forest (Stillman, Siegel, Wilkerson, Johnson, Howell, et al., 2019). In short, pyrodiversity can help some species (e.g., vagile terrestrial species) to complete their life cycles by accommodating habitat needs of different life stages (Stillman, Siegel, Wilkerson, Johnson, & Tingley, 2019).

Understanding how patterns produced by future fires will affect habitat diversity in future is an important research question for conservation biologists. One hypothesis is that the grain of interspersed nonforest patches within the forest matrix is an important controller of wildlife diversity and that fire regimes producing landscape patterns with the “right” grain for the largest number of species will have high conservation value.

## 6 WILDFIRE EFFECTS ON AQUATIC HABITAT

**FIGURE 2:**
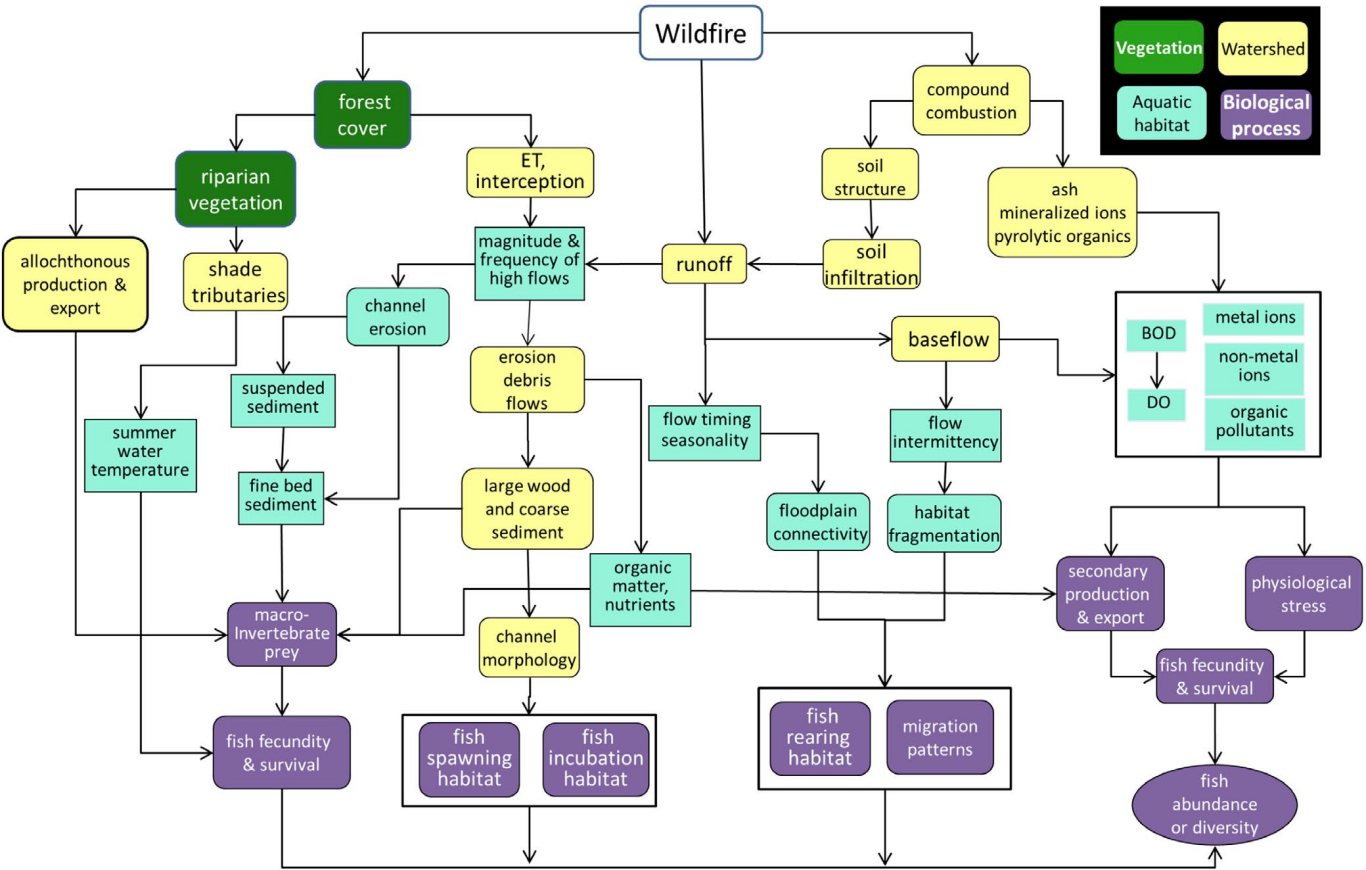
Diagram showing direct and indirect effects of wildfire on aquatic populations mediated by habitat via hydrologic, geomorphic, vegetative and biochemical pathways. BOD, biological oxygen demand; DO, dissolved oxygen; ET, evapotranspiration. Adapted from Paul, M.J., S. LeDuc, M.G. Lassiter, L.C. Moorhead, P. D. Noyes, and S.G. Leibowitz

In aquatic systems, aquatic productivity is stimulated by short‐ to midterm pulses of solar energy (from loss of shading) and allochthonous inputs from riparian growth or debris flows after fires (Minshall et al., 1989). Wildfire disturbance modifies physical habitat in streams and rivers through a number of pathways (Figure 2). The most immediate influences of wildfire are changes in stream temperature, water chemistry, and the erosion and deposition of ash and fine sediment from hillslopes (Minshall et al., 1989). Longer‐term influences are mediated by the intensity of postfire precipitation and involve episodic debris flows that deliver larger materials to river networks, such as large wood and coarse sediment, particularly in areas of steeper terrain (Miller et al., 2003).

### 6.1 Wildfires influence aquatic habitat through changes in temperature

Stream temperature shows responses at immediate, midterm, or longer‐term time scales following wildfire (Minshall et al., 1989). Immediate heating of very small streams occurs during high‐severity wildfires (Hitt, 2003). In some cases, smoke from fires may moderate temperatures by reducing short‐wave solar radiation (David et al., 2018), a primary driver of heat budgets in smaller streams (Caissie, 2006). Studies of postfire stream temperature have detected changes over a longer (>10 year) duration following debris flows that remove riparian vegetation (Dunham et al., 2007), whereas other systems exhibit influences that are detectable for only a few years (Schultz et al., 2017). Koontz et al. (2018) found similar responses to wildfires across the Pacific Northwest. Thermal fire refugia are promoted by groundwater inputs, riparian shading (Ebersole et al., 2003; McCullough et al., 2009), and by topographic variation leading to smoke shading (Downing et al., 2021).

### 6.2 Wildfires influence aquatic habitat through changes in water chemistry

Wildfires influence physical and chemical water quality in streams that drain burned catchments (Figure 2) (Hohner et al., 2019; Rust et al., 2018; Smith et al., 2011). Wildfires release minerals from the soil, stimulating primary production (Perera & Buse, 2014). Fire severity mediates the delivery of dissolved and total metals in streams (Abraham et al., 2017). High‐severity fires remove all vegetation and consume organic matter on the surface, whereas low‐severity fires leave some trees with live foliage intact (Keeley, 2009). Dissolved metals were significantly elevated in streams draining 20%–33% of burned watersheds in the western United States (Rust et al., 2018).

Vegetation recovery mediates the recovery time of water chemistry. Concentrations and loadings of dissolved and total metals increase dramatically after high‐severity fires, and subsequent storms can continue to influence streams until vegetation recovers, after which erosion abates along with mobilization of nutrients and metals bound to particulates (e.g., Rust et al., 2019). Water‐quality impacts from fire can be short‐term, lasting 1 to 3 years, or in some cases changes may be detectable for up to 10 years (Rust et al., 2018). Concentrations (loadings) of dissolved and particulate nitrogen and phosphorus remained elevated for at least five years after for 18%–38% (30%) of fires in the western United States (Rust et al., 2018). Persistent changes in water‐quality occurred where burn severity was high, where prefire soil organic matter was high, and where vegetation was slow to recover (Rust, Saxe, et al., 2019). For larger rivers, mixing of plumes with elevated levels of sediment, metals, and other constituents from wildfires in different tributary basins can put stress on downstream water‐treatment facilities (Emmerton et al., 2020).

### 6.3 Wildfires influence aquatic habitat though debris inputs and changes in sediment

Severe fires can alter soil structure and increase hydrophobicity, reduce soil infiltration, and increase runoff and erosion (Figure 2). Immediately postfire, ash and significant volumes of fine sediment are transported through watersheds and deposited downstream (Murphy et al., 2019; Reneau et al., 2007). Subsequent rainstorms continue to cause erosion within burned areas and mobilize river sediment (Moody et al., 2013; Rust, Saxe, et al., 2019). Consequently, streams draining burned areas experience elevated levels of suspended sediment and turbidity after wildfire (Moody & Martin, 2009; Rust, Randell, et al., 2019). Influxes of larger material (e.g., boulders and large wood) form pool and riffle sequences where channels are laterally constrained and more‐sinuous channel where they are not (Benda et al., 2004; Sedell et al., 2015). Large pools can develop upstream of confluences that experience substantial postfire sediment deposition; downstream, sediment influxes produce floodplains and terraces (Benda et al., 2004). On longer time scales, these geomorphic processes slow water and increase biotic resistance by creating refuge habitat (Reeves et al., 1995).

Wildfire‐mediated fluxes vary with topology. Historically, wildfires in the western United States tended to occur in forested headwater catchments, for example, in the Yellowstone fires (Minshall, 2003) and wildfires in New Mexico, United States (Frenette et al., 2019). Disturbances tend to have a larger impact in headwaters, and these impacts are attenuated downstream (Benda et al., 2004). Higher severity fires in steep terrains are more likely to produce debris flows (Cannon et al., 2010; Staley et al., 2017) that can deliver large wood (Zelt & Wohl, 2004), sediment (Arnold et al., 2017), pyrogenic carbon, and nutrients to streams (Cotrufo et al., 2016; Rust et al., 2018). Wildfire severity is also a key predictor of debris‐flow risk in the western United States (Gartner et al., 2008; Miller et al., 2003; Staley et al., 2017). Higher severity fires alter soil hydraulic properties and expose bare soil, which tends to increase delivery of water and sediment to rivers following precipitation events (Melatunan et al., 2009; Moody & Martin, 2004; Robichaud et al., 2019).

## 7 WILDFIRE EFFECTS ON AQUATIC FAUNA

Fire disturbance has negative effects on aquatic fauna in the short‐term, that is, for the months immediately following fire disturbance (Earl & Blinn, 2003). Pulse inflows of ash into streams suffocate aquatic biota by stimulating decomposition and lowering dissolved oxygen (Lyon & O'Connor, 2008; Whitney et al., 2015) (Figure 2). During the initial “acute” phase, and intermittently after postfire storms, suspended solids have negative effects on primary and secondary production. However, the longer‐term effect of postfire allochthonous inputs of carbon and nutrients is to stimulate production of aquatic invertebrates (Jackson et al., 2012). In one study, densities of benthic macroinvertebrates remained higher for 15 to 20 years before returning to levels found in unburned catchments (Scrimgeour et al., 2001). In a boreal headwater stream, mayflies and caddisflies increased in drift for more than ten years after fire (Musetta‐Lambert et al., 2019).

Regrowth of riparian vegetation is one factor that determines whether and at what rate aquatic communities will recover. Burning of the riparian canopy increases solar radiation in small streams, stimulating primary production. Aquatic macroinvertebrate biomass increased following severe fire in Idaho that opened stream canopies (Minshall et al., 2001; Rugenski & Minshall, 2014) In one study, linked aquatic–terrestrial ecosystems recovered after 5 to 10 years (Jackson et al., 2012; Malison & Baxter, 2010b). In another case, the riparian canopy did not fully recover twenty years after the 1990 Dude fire, which burned over 10,000 ha in Arizona (Leonard et al., 2017). Because of the significant restructuring of the headwater channels, the macroinvertebrate community did not recover to its prefire abundance or diversity, and efforts to re‐establish Gila trout were unsuccessful, likely due to higher summer stream temperatures (Leonard et al., 2017). Yet, farther south, in the upper Rio Grande, aquatic macroinvertebrates and salmonid fishes recovered within five years after a large fire with greater than 60% high severity (Alhassan et al., 2019).

### 7.1 Aquatic invertebrate responses to wildfire are mediated by fire regime

As in terrestrial ecosystems, the effects of wildfire on stream‐riparian ecosystems and their aquatic fauna are strongly mediated by fire severity and the percent of catchment burned (Minshall et al., 2001). Whether aquatic communities recover to a prefire state depends on fire severity, size (areal extent), and frequency. Species composition may not recover to its prefire state if hydrologic disturbances postfire are repeated and prevent recolonization (Mata et al., 2012; Vieira et al., 2004) or if significant structural modifications to the catchment or streams occur. Following more severe fires, channel‐modifying debris flows and other direct effects can directly kill aquatic life and reduce the richness of macroinvertebrate communities in the short‐term (Minshall, 2003; Rinne, 1996). Large, high‐severity fires may alter catchments and produce debris flows that substantially alter stream channels in ways that delay regrowth (Leonard et al., 2017). Widespread high‐severity fires can also cause mortality of crayfish and other crustaceans (Silva et al., 2020).

Allochthonous resources for aquatic macroinvertebrates and fish are influenced by fire severity (Jackson & Sullivan, 2009). Where in‐channel habitat is not substantially altered, postfire increases in phosphorus and other nutrients can lead to increased algal production, macroinvertebrate density and diversity, and fish growth (Emelko et al., 2016). Streams in Idaho draining areas that burned with high severity exported more emerging adult insects to riparian consumers than streams draining unburned areas and those burned at intermediate severity. Reaches draining high‐severity burns supported a higher proportion of r‐selected species (Opportunists, Figure 4) than low‐severity burns (Malison & Baxter, 2010a).

Recovery dynamics are also driven by the areal extent of patches of severe wildfire. Following the 1998 Yellowstone fires (Minshall et al., 1997), some components of the aquatic community had recovered to their original state within a decade, whereas others did not (Minshall, 2003). Aquatic invertebrates did not return to their original states in stream reaches where burns exceeded 25 and 50% of contributing watersheds (Minshall, 2003; Minshall, Royer, et al., 2001).

### 7.2 Aquatic invertebrate responses to wildfire are mediated by life history traits

Because the impact of wildfire disturbances in aquatic systems is carried by postfire floods, we expect traits that confer resistance to flooding to be relevant to burns. These traits include a streamlined shape, adaptations for clinging to substrate, and having at least two stages outside the stream (Chiu & Kuo, 2012). Species with higher elasticity include opportunists with a short life cycle and habitat generalists, “movers,” that seek refuge and later recolonize (Figure 4) (Berg et al., 2010; Chiu & Kuo, 2012). More‐frequent disturbances by sediment influxes favor invertebrate taxa that are short‐lived or multivoltine (having two or more broods per year) (Buendia et al., 2013) and that burrow (Bury, 2004). Burrowing aquatic invertebrates (e.g., mussels) seek refuge in sediment (Stayers, Figure 4). Aquatic invertebrates also have traits, such as mobility, that promote recovery by finding refuge in areas of river protected from silt deposition (i.e., behind structures) and recolonizing following disturbance (Li et al., 2016) (Movers, Figure 4).

Immediately after a fire, fine‐sediment deposition exposes less‐mobile species and life stages with low spatial elasticity to high risk (Figure 4). For example, sessile, filter‐feeding mussels are vulnerable to influxes of fine sediment following wildfire (Santos et al., 2015). To recover, some fraction of mussel beds must be located in nondepositional refugia. For many mussel species, colonization of previously disturbed reaches requires infecting host fishes with glochidia (larvae) that are transported to colonize other reaches. Depending on the distribution of the mussel species relative to the spatial extent of wildfire effects and the availability of fish hosts, the effects of sediment disturbance could be short‐term or lead to long‐term extirpation from affected reaches.

Community responses are not restricted to the stream. Wildfire can stimulate the flux of aquatic prey to terrestrial habitats and increase riparian consumers (Malison & Baxter, 2010b). These effects on benthic invertebrates subsequently affect fishes and wildlife that feed on them, both in aquatic and terrestrial ecosystems. Recovery occurs as a result of bottom‐up faunal succession and food web assembly, which is influenced by time since disturbance.

### 7.3 Fish respond to areal extent, severity, and frequency of wildfire

Watersheds support spawning of migratory and resident fish species that exist in metapopulations. Here, we use the term “metapopulation” in the broad‐sense that includes patchy populations (Harrison, 1991) that experience extirpation and later recolonize when facing a nonstationary future climate and human‐modified landscapes. Plasticity in spatial life histories of western North American fishes likely confers resilience to watershed disturbances, including fire (Dunham et al., 2003; Reeves et al., 1995). Pacific salmon and steelhead, are anadromous species that spend their adult lives in marine environments and return to natal rivers to spawn. Populations are protected by the fraction at sea that avoid immediate impacts from wildfires. Furthermore, spawning fish can subsequently return to their natal streams or stray into non‐natal streams to recolonize impacted habitats (Reeves et al., 1995). Populations following a “Movers” spatial life history (Box 1) facilitated community recovery by recolonizing streams after wildfire disturbance in the Boise River system (Rieman et al., 1997). Migratory individuals were outside of a headwater system when a severe wildfire apparently extirpated all remaining fish. Returns of migratory bull trout later enabled the local population to persist in the face of disturbance (Rieman et al., 1997). Rainbow trout (*Oncorhynchus mykiss*) in the Boise River, Idaho persisted in severely burned tributary systems, including those that have experienced substantial channel reorganization (Dunham et al., 2007; Neville et al., 2009). These species show a contrast in their life history responses to wildfire disturbance: rainbow trout “persist in place,” whereas bull trout “shift in space” (see Thurman et al., 2020). Those that “persist in place” may experience elevated temperatures and incur higher energetic costs following severe wildfires (Beakes et al., 2014).

The timing of wildfires and subsequent floods relative to reproduction (i.e., fall versus spring spawning species) may mediate how populations are affected, especially for migratory species. An influx of spawning gravels and sediment could be beneficial if floods wash away fine sediments before spawning (Kondolf et al., 1996). However, large influxes of fine sediment during spawning can bury spawning gravels and fill pore spaces, reducing the survival of early salmonid life stages (Greig et al., 2005; Louhi et al., 2011). In the longer term, deposition of large wood and boulders may buffer downstream channels against sediment deposition. Large woody debris creates pools and structural complexity that benefit salmonids (Flitcroft et al., 2016) by maintaining a mixture of reaches in aggrading and degrading states (Reeves et al., 1995). Whereas the successional preferences of terrestrial wildlife have been documented, including preferences for years since burn (Nelson et al., 2008), we are not aware of such information for aquatic biota.

**FIGURE 3:**
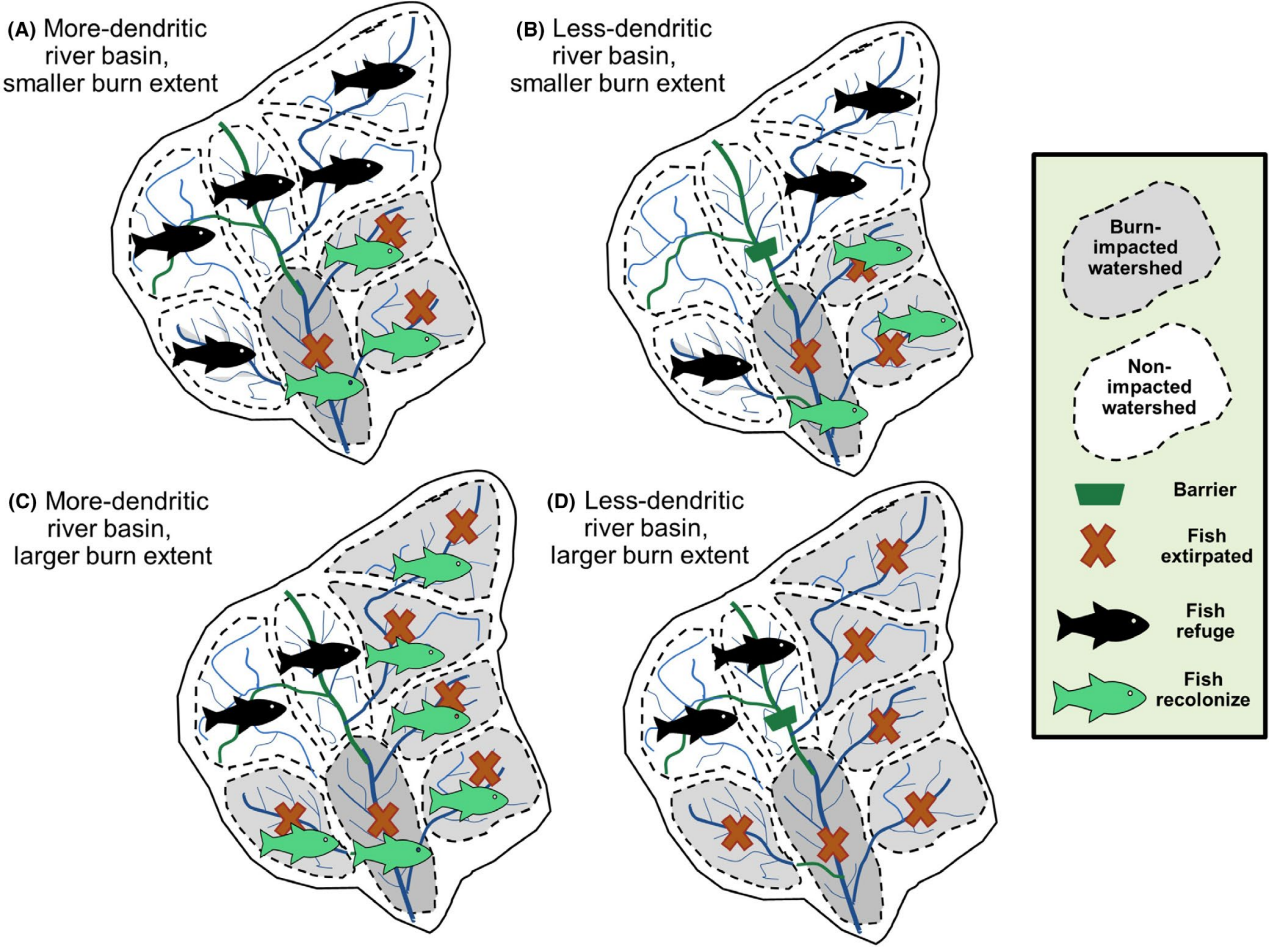
Diagram of potential for population recovery in a river basin with tributary basins. The four cases illustrate that subpopulations are less likely to re‐establish when widespread severe fires extirpate many subpopulations (c, d) than when fewer watersheds are affected (a, b). Secondly, watersheds with high connectivity (many tributaries and few barriers – a, c) are more likely to have unburned refuge areas from which fish and other stream‐constrained biota can recolonize than those with fewer accessible tributaries (b, d)

**FIGURE 4:**
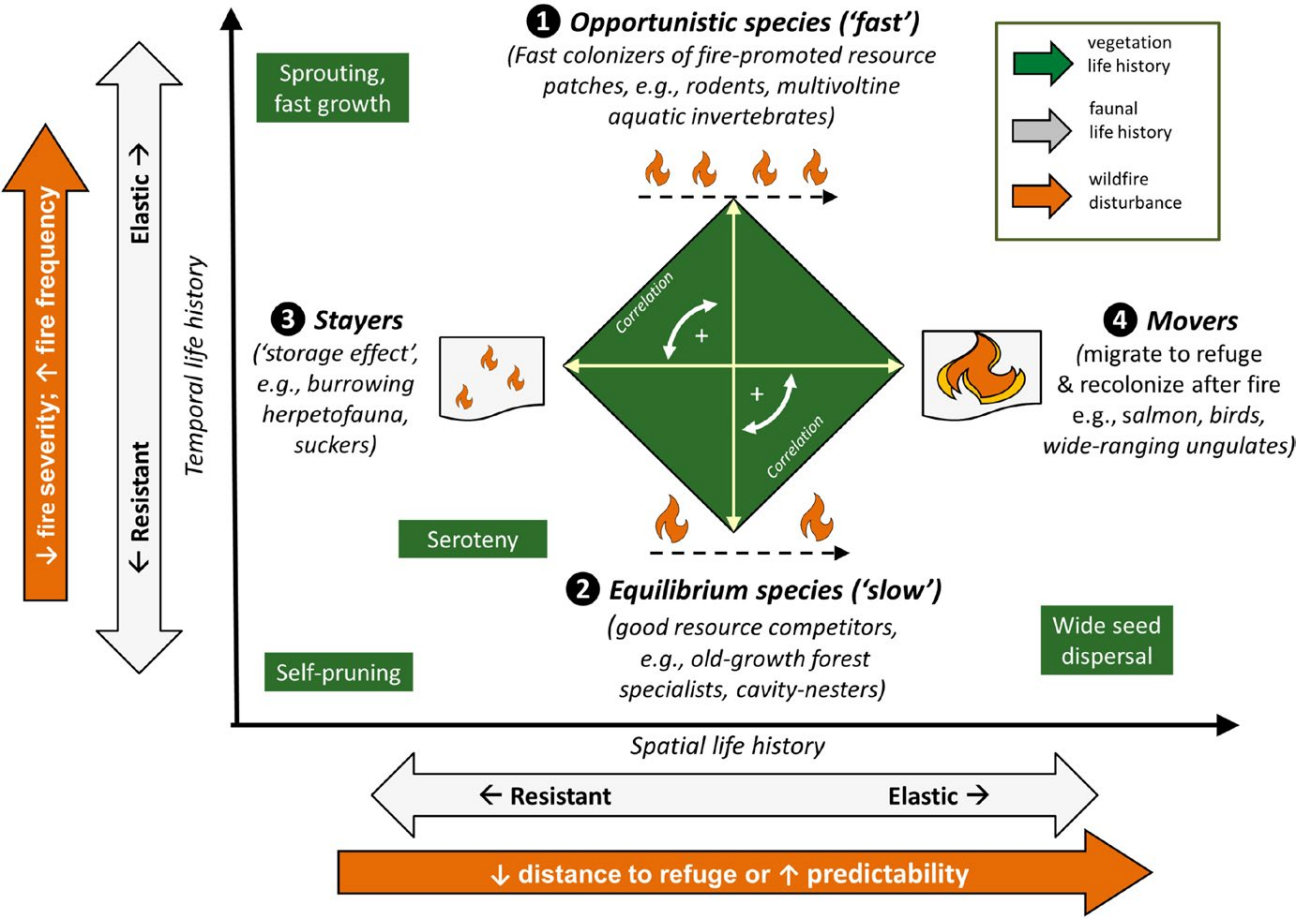
Species life history traits in time (*y*‐axis) and space (*x*‐axis) reflect the wildfire regimes to which they are adapted. The framework presented differs from triangular ones (Winemiller & Rose, 1992) in that trade‐offs and correlations are not imposed and a spatial dimension is added. (1) Opportunistic species show rapid responses to habitat disturbance and dominate habitats subjected to relatively frequent and unpredictable patterns of disturbance. (2) Equilibrium‐type species are favored in stable habitats experiencing less exposure to abiotic stress (e.g., fire refugia). Defined with respect to spatial life history, we propose two endpoints: (3) Stayers persist through disturbance using “resistant” strategies – that is, long‐lived highly fecund species with intermittent reproductive bouts that can withstand fire by sheltering in place. (4) Movers are populations that recover through high mobility

Recovery of a local fish community to its prefire state does not always happen following wildfires. Fire‐related extirpations of fish populations from stream reaches have been observed, and long‐term recovery depends on successful recolonization by populations in less‐affected waterbodies (Dunham et al., 2003). When high‐intensity wildfires impact a significant part of a river network, fish populations and other aquatic biota may take longer to recolonize or fail to re‐establish (Figure 3). Following several wildfires in east‐central Arizona, fish populations were extirpated when 50% of the upstream watershed area burned at moderate‐ to high‐severity, causing extensive channel infilling by debris (Long, 2008). In a study of fish recovery following wildfire disturbance in the Gila Basin, NM, the presence of large tributary and valley reaches draining unburned areas served as an important source for recolonization (Meddens et al., 2018; Whitney et al., 2016). Gido et al. (2019) found that resistance of fish communities to drought and wildfire events in desert streams was determined by the severity of disturbance, whereas the recovery rate (elasticity) was determined by the ability of fish populations to rebound from severely depressed numbers (opportunists, Figure ) or to immigrate from nearby refuge populations (movers, Figure 4). Because they are more isolated than mainstem reaches, tributaries were recolonized two years later than mainstem reaches (Gido et al., 2019). Stream fragmentation has contributed to local fish extirpations that might have been avoided if the severity and spatial footprint of disturbance was reduced or if populations had access to refuge (Gido et al., 2019; Whitney et al., 2017). Similar patterns have emerged in salmonid populations in forested watersheds of the US Pacific Northwest (Falke et al., 2015).

We hypothesize that river networks with a higher density of connected tributaries will provide higher resilience to aquatic populations than networks with fewer tributaries or more‐isolated tributaries (e.g., those blocked from the mainstem by poor water quality, waterfalls, dams created by humans, debris, or beavers, or other barriers) (Terui et al., 2018) (Figure 3). For similar reasons, larger, undammed watersheds have been shown to support more stable fisheries than smaller watersheds or those with obstacles to movement (Moore et al., 2015). Yet, waterbodies, including artificial ones, may interrupt sediment flows and reduce short‐term impacts on downstream reaches. Refuge is an important consideration when evaluating the resilience of aquatic communities to wildfire disturbance (Berryman & Hawkins, 2006).

## 8 CLIMATE ADAPTATION PLANNING FOR FORESTS OF WESTERN NORTH AMERICA

Climate change is increasing the frequency and size of severe fires. A concern is that larger fires will increase habitat homogeneity (Vanbianchi et al., 2017) and lead to shifts away from coniferous forests, potentially harming taxa that depend on them. Vegetation shifts away from coniferous forest in response to shifting wildfire regimes under trending climate are inevitable. The climate‐driven boundaries of primary vegetation types (forest‐shrubland‐grassland) will shift, and coniferous forest will become more fragmented. It is unlikely that losses of coniferous forest will be averted under drier, warmer conditions with reduced snowpack (Schoennagel et al., 2017). At low elevations, thresholds in soil surface temperature and moisture were passed during the past 20 years that reduce postfire tree regeneration at drier sites (Davis et al., 2019). Meanwhile, loss of snowpack is increasing wildfire risk at higher elevations (Gergel et al., 2017).

Climate changes are causing migrations of animal species along latitudinal and altitudinal gradients (Pecl et al., 2017). Poleward migrations have occurred at rates between 11 and 16.9 m per decade (Chen et al., 2011). Along elevational gradients, animal populations are expected to move to higher elevations, but they may be squeezed between upward shifts in suitable climate and suitable wildfire regimes at lower elevations. Evidence suggests that cold‐water fishes will shift to higher elevations in response to warming climate (Eby et al., 2014; Jager et al., 1999), and similar predictions have been made for terrestrial wildlife (Furnas, 2020). Increased wildfire impacts on conifers at high elevations may therefore restrict the ability of species to adapt to other climate‐related shifts by migrating to higher elevations.

Climate adaptation plans for forest management typically consider options to increase resistance (forestall impacts and protect highly valued resources), options to increase elasticity (improve the capacity of ecosystems to recover after disturbance), and options that facilitate ecosystem transitions to new conditions when they are inevitable (Millar et al., 2007).

### 8.1 Managing for forest resilience

Climate change has been shown to be an important anthropogenic driver of shifts in wildfire (Abatzoglou & Williams, 2016), and scientific guidance exists on actions needed to slow or avert further shifts toward warmer, drier conditions, such as transitioning to a low‐carbon economy, are well known (IPCC, 2014). Wildfire creates a positive feedback loop by adding carbon to the atmosphere and changing the earth's radiative budget (Liu et al., 2019). Satellite data revealed that warming persisted after fire at low latitudes, whereas wildfires in boreal forests caused warming for five years, followed by a small cooling effect (Liu et al., 2019).

In many western forests of North America, fire‐suppression policies have exacerbated the effects of climate by increasing the density of trees and fuel build‐up. Fire suppression has created a positive feedback leading to shifts in vegetation and fire regimes (Calkin et al., 2015). For example, fire suppression in California has allowed dense stands of conifers to outcompete hardwoods and chaparral (White & Long, 2019). Western forests were more resilient to wildfire before the era of fire suppression and restoring presuppression states may slow ecosystem shifts associated with climate (Hessburg et al., 2015).

Forest treatments, including prescribed burning and thinning, can be used to increase resilience by generating mosaic landscapes with patches of conifers and early seral forest or grassland (Kalies & Yocom Kent, 2016; Whitney et al., 2016). Mosaic landscapes are more resistant to large, stand‐replacing fires and reburns because nonconifers interrupt fuel connectivity (Abella et al., 2007; White & Long, 2019). Because treatment is not feasible at large scales, strategic deployment is important, raising questions about priorities. Should treatments focus near or away from major boundaries between ecotypes? Should treatments focus on drier, more‐vulnerable forests, that is, those in dry topographic positions (Halofsky et al., 2020; Hankin et al., 2019) or on wetter sites, including less‐vulnerable riparian zones, old‐growth refugia, and boreal peat forests? Is landscape heterogeneity a strategic goal, that is, treating patches within large swaths of dense, homogeneous forest (Hessburg et al., 2015)? Can aquatic ecosystems be protected by strategic risk spreading across nested watersheds in river basins?

The spatial scale at which positive feedback between wildfire/climate and vegetation shifts can efficiently and effectively be interrupted is central to these decisions. For example, are there sizes and arrangements of patches that lead to a quasi‐stable state (resting point) under expected rates of climate change? Research is needed to characterize conditions that support persistent mixed forests (e.g., refuge areas with topographic variation) (Downing et al., 2021). Opportunities for increasing resilience through postfire management also exist. When stand‐replacing fires consume tall conifers, meadows or hardwood groves can be permitted to reemerge in gaps (Boisrame et al., 2017; Hessburg et al., 2019). Postfire, active revegetation may help to speed revegetation of uncharacteristically large burns where seed sources have been compromised. Such interventions can slow the transition away from coniferous forests as climate changes and wildfires become larger and more severe (Adams, 2013).

### 8.2 Managing for faunal resilience

Forest restoration is often focused on vegetation; restoring trophic function has not been a prominent goal (Fraser et al., 2015). Looking beyond the trees and forests to fauna, a future transition to mixed conifer/nonconifer landscapes should benefit fauna that are less dependent on coniferous forests (White & Long, 2019). Restoration options that reduce fire frequency, and those that produce interspersed refuge areas (unburned or burned at lower severity), will increase the availability of snags and other structures that provide cover and, therefore, accelerate recolonization by cover‐dependent wildlife (Fisher & Wilkinson, 2005). Retaining large, live residual green trees increase elasticity of a coniferous forest matrix by reseeding surrounding areas and provide mast for wildlife (Hessburg et al., 2015).

Late‐successional (“old‐growth”) forests are increasingly rare, yet they support threatened or endangered species with specialized habitat requirements (Dellasala & Hanson, 2019; Jones et al., 2016; Wan et al., 2019). Fortunately, these forests, with cooling from multilayer canopies and larger, hardier trees, tend to burn at lower severity (Lesmeister et al., 2019), and permitting fires in less‐vulnerable habitats or under cool, wet conditions has been suggested (Reilly et al., 2017). Research is needed to assess whether the wildfire‐resistant features of old‐growth forests and associated wildlife will be sufficient to avoid tipping points (loss of old‐growth dependent wildlife communities), or whether interventions can prevent further loss of habitat. If more severe fires occur more frequently, we can expect species that require these forests to be impacted by increased fragmentation and habitat loss (Spies et al., 2019). For example, under future climate, woodland caribou may face competition from other ungulates (e.g., deer) as grasslands replace old‐growth forest that provide winter forage (Barber et al., 2018).

Decision support tools for biological conservation have been developed in Australia, where optimal configurations of burn histories were designed for an area supporting multiple species at risk based on established preferences for landscape configurations (Kelly et al., 2017) and time since fire (Nimmo et al., 2013). Targeted fuel treatments in surrounding areas can potentially protect rare, threatened habitats. For example, a California study found that landscape heterogeneity resulting from the spatial arrangement of fuel treatments buffered populations of Northern flying squirrels against wildfire impacts (Sollmann et al., 2016). Another study that simulated thinning in old‐growth forest found that indirect positive effects of habitat heterogeneity outweighed the direct negative effects of thinning on fishers (Westerling et al., 2006).

Because traits related to (re)colonization promote recovery following disturbance (Movers, Figure 4), it is important to investigate whether different species can safely travel through different habitat types. The ability of animals to adapt to climate warming and changing disturbance regimes can be facilitated by removing barriers (Murphy et al., 2020). For example, fencing that blocks wildlife movements on land (Sitters & Di Stefano, 2020) and poorly designed culverts and crossings that block fish movements in streams (Neville et al., 2009, 2016). Forest restoration (e.g., riparian buffers) or treatments can create corridors that help some species to colonize new habitat following displacement. In freshwater ecosystems, similar restoration options exist for promoting resilience. For example, access to diverse aquatic habitats (e.g., tributaries, floodplains, mainstems) can add resilience to freshwater assemblages against future increases in wildfire size, severity, and frequency (Bisson et al., 2003; Dunham, Rieman, et al., 2003; Millar et al., 2007). Recovery plans for species listed under the US Endangered Species Act recognize this by including spatial diversity and connectivity as two of four criteria required to determine whether distinct population units have recovered (McElhany et al., 2000).

### 8.3 Managing ecosystem transitions

Restoring past disturbance regimes (historical fire regimes) has been promoted as a conservation priority in North America (Freeman et al., 2017). The assumption is that fauna adapted to historical conditions will not be able to track fast changes. Yet, considerable hubris is needed to claim that we can reconstruct past fire regimes. These are not well known and may not produce resilient ecosystems when facing a nonstationary future climate and human‐modified landscapes (Freeman et al., 2017; McWethy et al., 2019). Species adaptation is aided by predictability, by maintaining large enough spatially distributed populations to conduct natural genetic experiments, and by facilitating migration. When ecosystem shifts are inevitable, the risk of faunal extirpations can be minimized by interventions that slow the rate of transition and by managing fire disturbance to promote negative feedbacks.

If transitions are gradual, species may be able to colonize areas that become newly suitable. This can be facilitated by identifying habitat in fire refuges and removing obstacles that help animals to track them (Meddens et al., 2018; Meigs & Krawchuk, 2018). Forested riparian corridors play a special role by providing refuge from wildfire for terrestrial wildlife (Pettit & Naiman, 2007) and thermal refuge for aquatic biota (Ebersole et al., 2003; Downing et al., 2021). In some cases, conservation of species may require translocation or other active interventions to establish spatial diversity among weakly linked populations (Stein et al., 2013). Reducing other threats can also help species to persist (Keeley & Brennan, 2012).

## 9 CONCLUSION

We are witnessing compositional changes in western North America's forests in response to climate change and past wildfire suppression. Climate scenarios predict that changes are inevitable under current projections of greenhouse‐gas emissions, and could potentially lead to a “sixth extinction” (Barnosky, 2015). Western North America includes at‐risk biodiversity hotspots such as the relic pine‐oak montane woodlands of the Madrean archipelago (Davis et al., 2015). Here, we reviewed a significant literature describing interactions among climate, wildfire regimes, vegetation, and aquatic and terrestrial biota.

Our goal was to understand how to minimize disruption of terrestrial and aquatic biota. Although the path to slowing the transition away from forest is fairly well illuminated, the path to protecting wildlife is much less clear. We developed a life history framework that identified two strategies to enhance resilience along the spatial axis of Figure 4 (Box 1). First, to maintain residual structural features and protect ecosystems rarely burn and provide wildlife refuge (Meddens et al., 2018), and second, to create safe corridors that facilitate species use of refuge and ability to recolonize (enhancing population‐scale elasticity). Research to quantify spatial relationships between resilience‐enhancing habitats potentially important to wildlife (e.g., Robinson et al., 2013) and wildfire regimes is needed in western North America. Furthermore, relevant metrics of access to refuge should be calculated within boundaries (e.g., watersheds, road networks) that constrain faunal responses to fire.

Improving the resilience of animal communities in the face of future climate is not just an academic exercise; some indigenous Americans depend on these populations (Nelson et al., 2008). Additional research is needed to identify self‐sustaining interventions that minimize disruption to western North American forest denizens as their habitat changes.

## CONFLICTS OF INTEREST

We have no conflicts of interest.

## AUTHOR CONTRIBUTION

**Henriette I. Jager:** Conceptualization (equal); Funding acquisition (equal); Project administration (lead); Supervision (lead); Visualization (equal); Writing‐original draft (lead); Writing‐review & editing (lead). **Jonathan W. Long:** Conceptualization (equal); Funding acquisition (equal); Resources (equal); Visualization (supporting); Writing‐original draft (equal); Writing‐review & editing (equal). **Rachel L. Malison:** Writing‐review & editing (supporting). **Brendan P. Murphy:** Writing‐original draft (supporting); Writing‐review & editing (supporting). **Ashley Rust:** Writing‐original draft (supporting); Writing‐review & editing (supporting). **Luiz G. M. Silva:** Writing‐review & editing (supporting). **Rahel Sollmann:** Writing‐review & editing (supporting). **Zachary L. Steel:** Conceptualization (supporting); Visualization (supporting); Writing‐original draft (supporting); Writing‐review & editing (supporting). **Mark D. Bowen:** Writing‐original draft (supporting). **Jason B. Dunham:** Funding acquisition (equal); Writing‐original draft (equal); Writing‐review & editing (equal). **Joseph L. Ebersole:** Funding acquisition (equal); Visualization (equal); Writing‐original draft (equal); Writing‐review & editing (equal). **Rebecca L. Flitcroft:** Writing‐original draft (supporting); Writing‐review & editing (supporting).

## Data Availability

Not applicable.

## APPENDIX 1

**TABLE A1:** Life history framework for wildlife response to wildfire disturbance adapted from (Winemiller & Rose, 1992)

Life‐history	Disturbance	Demography	Resilience strategy	Examples
Equilibrium	Infrequent and/or fine‐scale disturbance	‘Slow’ life history (K‐selected): High age at maturity High juvenile survivorship (e.g., parental care)	Use of relatively stable, undisturbed habitats (e.g., wildfire refuge); in freshwater networks, locations sheltered from sediment flows	Old‐growth forest specialists (e.g., cavity‐nesting birds relatively protected from predators, mammals with poor gap‐crossing ability) Hyporheic habitat in streams; non‐depositional habitats
Opportunistic	Unpredictable, frequent, small‐scale disturbance	‘Fast’ life history (r‐selected); Low age at maturity Small body size and # offspring/birth event, but may reproduce multiple times per season Low juvenile survivorship (little or no parental care)	Ability to quickly colonize recently burned habitat (high elasticity)	Many rodents Multivoltine aquatic invertebrates
Stayers	NA	Non‐migratory	Spatial traits that confer resistance to wildfire and low vulnerability to fire (e.g., burrowing, use of nearby refuge, demographic storage effect)	Burrowing herpetofauna Most small mammals use underground tunnels or paths under forest litter. Aquatic invertebrates that burrow
Movers	Large‐scale disturbance	Migratory	Spatial traits that confer elasticity, such as the ability to migrate to wildfire‐generated habitat.	Large wide‐ranging ungulates that track mid‐ successional habitat Volant mammals (e.g., bats, flying squirrels) Volant birds Pacific salmon, steelhead, sturgeon, bull trout, lamprey

Note

To emphasize the importance of spatial life‐history adaptations to wildfire disturbance, we included the spatial extent of disturbance as one dimension (Figure 4). Spatial life history characteristics are important to consider because they distinguish species with strategies at the resistant versus elastic end of the spectrum describing response to fire disturbance. Migratory species exemplify elasticity, capable of responding to disturbance trends and climate‐driven shifts in habitat through movement (Harsch et al., 2014) (Movers in Figure 4).
